# In-hospital Outcomes and Arrhythmia Burden in Patients with Obstructive Sleep Apnea and Heart Failure with Preserved Ejection Fraction

**DOI:** 10.19102/icrm.2022.130602

**Published:** 2022-06-15

**Authors:** Jashan Gill, Chunyi Wu

**Affiliations:** ^1^Department of Medicine, Rosalind Franklin University of Medicine and Science, North Chicago, IL, USA; ^2^Department of Medicine, Northwestern Medicine McHenry Hospital, McHenry, IL, USA; ^3^Department of Medicine, University of Michigan, Canton, MI, USA

**Keywords:** Atrial fibrillation, diastolic heart failure, heart block, sick sinus syndrome, sleep-disordered breathing

## Abstract

Patients with obstructive sleep apnea (OSA) have an increased risk for arrhythmias compared to patients without OSA. However, data quantifying the risk of inpatient complications in patients with heart failure with preserved ejection fraction (HFpEF) are lacking. We sought to compare inpatient outcomes and the occurrence of arrhythmias in patients with HFpEF with and without OSA, respectively. Furthermore, we compared the prevalence of arrhythmias with nocturnal continuous positive airway pressure (CPAP) therapy. We performed a retrospective study using the National Inpatient Sample from 2016–2018 to identify patients with HFpEF with and without OSA. Propensity score matching, adjusting for age, gender, race, hospital characteristics, income, and comorbidities, was used to select matched samples between both groups. From 2016–2018, 127,773 hospitalizations with HFpEF were identified; among these patients, 20% had OSA. Nocturnal CPAP was utilized in 9% of these patients. Patients with OSA had a higher mortality rate, a longer duration of hospitalization, and greater medical costs. In addition, OSA was associated with higher incidence rates of atrial fibrillation, atrial flutter, premature depolarization, sick sinus syndrome, ventricular tachycardia, and atrioventricular block. Nocturnal CPAP was not associated with a lower arrhythmia incidence; however, there was a non-significant trend toward a lower cardiac arrest incidence. In conclusion, OSA in patients with HFpEF was associated with greater mortality, longer hospitalization stays, and higher medical costs relative to findings in patients without OSA. Furthermore, OSA was associated with tachyarrhythmias and bradyarrhythmias in HFpEF patients. Nocturnal CPAP was only utilized in 9% of patients, with no difference in arrhythmogenesis.

## Introduction

Heart failure with preserved ejection fraction (HFpEF) represents approximately half of all heart failure diagnoses worldwide, and its prevalence is increasing annually.^1,2^ Moreover, it is estimated that approximately 30% of men and 15% of women meet the diagnostic criteria for obstructive sleep apnea (OSA) in North America.^3,4^ Heart failure and OSA have considerable overlap as observational studies have shown that OSA is present in roughly 50% of HFpEF patients.^5^

OSA is characterized by recurrent intermittent upper-airway obstructive events, leading to hypoxemia, hypercapnia, and autonomic dysregulation.^6^ OSA has been associated with sudden cardiac death^7,8^ and a broad spectrum of cardiac rhythm disorders, with the most evidence being available for atrial fibrillation.^9,10^ However, the prevalence of arrhythmias in HFpEF patients with OSA has not been definitively established.^11–15^ Furthermore, studies have demonstrated a reduced atrial fibrillation burden with continuous positive airway pressure (CPAP) therapy.^16–18^ The data regarding CPAP therapy with other arrhythmias are preliminary but point toward reduced incidence rates of bradyarrhythmia,^11^ ventricular ectopy,^19^ and sudden cardiac death.^20^ However, there is a paucity of data from heart failure patients.

The purpose of this study was to compare the prevalence of cardiac arrhythmias in HFpEF patients with and without OSA, utilizing the National Inpatient Sample (NIS) database from 2016–2018. In addition, we sought to compare in-hospital mortality rates, the length of hospitalization, and medical costs of hospitalization among these patients. Finally, we examined the utilization of inpatient nocturnal CPAP in patients with OSA to assess whether the incidence of arrhythmias was decreased in these patients compared to patients who did not receive nocturnal CPAP.

## Methods

### Data source

The data for this study were drawn from the NIS database, the Healthcare Cost and Utilization Project, and the Agency for Healthcare Research and Quality (AHRQ).^21^ The NIS is the largest collection of all-payer data on inpatient hospitalizations in the United States. The dataset represents an approximated 20% stratified sample of all inpatient discharges from U.S. hospitals. The database provides de-identified information for each hospitalization. This information includes patient-level and hospital-level factors, such as patient demographic characteristics, primary and secondary diagnoses and procedures, AHRQ comorbidities, length of stay, hospital characteristics, and costs of hospitalization. National estimates can be calculated using the patient-level and hospital-level sampling weights that the NIS provides. For this study, data were obtained from the years 2016–2018.

### Study population and variables

The International Classification of Diseases, 10th revision (ICD-10), was used to report diagnoses and procedures in the NIS database. We identified patients hospitalized with HFpEF (ICD-10–Clinical Modification [CM] codes I530, I5031, I5032 and I5033) and OSA (ICD-10-CM code G4733). We excluded patients aged <18 years, those with a body mass index (BMI) of <29.9 kg/m^2^, and those with a pacemaker or an implantable cardioverter-defibrillator. Of the patients with HFpEF and OSA, patients who utilized nocturnal CPAP (ICD-10 Procedure Coding System code 5A09357) were also identified. In order to mitigate selection bias and control for patient and institutional imbalances, propensity scoring was used to select matched samples of HFpEF patients with OSA and those without OSA. The scoring was based on a multivariate logistic regression model, accounting for age, gender, race, hospital type, hospital region, hospital teaching status, median household income, and medical comorbidities (cerebrovascular disease, chronic kidney disease, chronic obstructive pulmonary disease, coronary artery disease, diabetes, hypertension, obesity, peripheral vascular disease, smoking, and valvular heart disease). Using 8- to 1-digit matching, we paired each diastolic heart failure admission with OSA with an admission without OSA.

### Study outcomes

The primary analysis of this study was a comparison of in-hospital mortality, length of hospitalization, and medical cost of hospitalization between HFpEF patients with OSA and those without OSA. The secondary analysis compared the prevalence of arrhythmias, including atrial fibrillation, atrial flutter, atrioventricular block, cardiac arrest, premature depolarization (atrial, ventricular, and junctional), sick sinus syndrome, supraventricular tachycardia (SVT), ventricular tachycardia, and ventricular fibrillation, in these patients. A subgroup analysis was also conducted in patients with HFpEF and OSA, comparing the incidence of these arrhythmias in those with and without inpatient nocturnal CPAP.

### Statistical analyses

All statistical analysis was performed using SAS Survey Procedures (SAS version 9.4; SAS Institute Inc., Cary, NC, USA). Statistical significance was defined by a 2-sided test with *P* < .05. The national estimates were calculated after accounting for the sample design elements (clusters, strata, and trend weights) provided by the NIS.^21^ Continuous variables were reported as weighted mean ± standard deviation (SD) values, and categorical variables were reported as weighted numbers (n) and percentages (%). The SDs of weighted means were estimated using the Taylor linearization method that incorporated the sample design. The Rao–Scott-modified chi-squared test was used to test the distribution difference for categorical variables, while a weighted Student’s *t*-test was used to analyze the normally distributed continuous variables. Variables that were not normally distributed were tested using the Wilcoxon rank-sum test. Multivariate logistic regression was used to estimate the odds ratio of in-hospital mortality and several outcomes after adjusting for patient demographics, hospital type, hospital region, hospital teaching status, median household income, and medical comorbidities.

## Results

### Population characteristics and comorbidities

**Figure 1:**
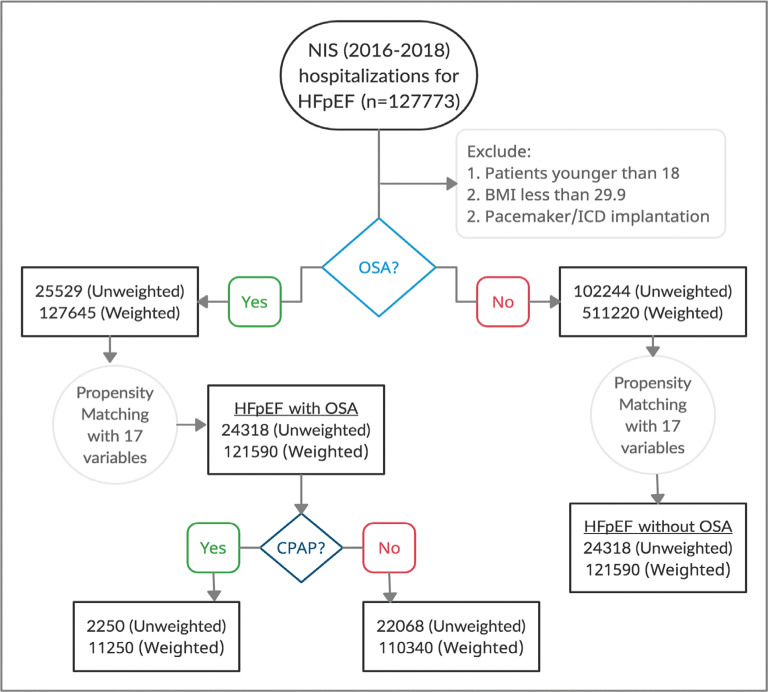
Study design flowchart. *Abbreviations:* BMI, body mass index; CPAP, continuous positive airway pressure; HFpEF, heart failure with preserved ejection fraction; ICD, implantable cardioverter-defibrillator; NIS, National Inpatient Sample; OSA, obstructive sleep apnea; SD, standard deviation.

The design of this study is presented in the flowchart in **Figure 1**. From 2016–2018, we identified a total of 127,773 unweighted hospitalizations with HFpEF; of these patients, 25,529 (20%) also had OSA. After applying propensity scores, nocturnal CPAP was utilized in 2,250 (9%) of these patients. Demographic and baseline characteristics of these patients before and after applying propensity matching are presented in **Table 1**. The mean age of the patients with HFpEF and OSA was 70 years, while that of those without OSA was 76 years. Of the patients with HFpEF, OSA was seen at similar rates in men (50%) and women (50%); however, in patients without OSA, there was a predominance of women (62%) with OSA. The racial distribution in patients with HFpEF and OSA was 73% Caucasian, 17% African American, 6.3% Hispanic, 1.1% Asian, 0.4% Native American, and 1.7% unspecified. The majority of patients were equally distributed in both groups; however, there was a trend toward an increased number of African Americans among patients with OSA. In patients with HFpEF and OSA, there were increased rates of chronic kidney disease, chronic obstructive pulmonary disease, coronary artery disease, diabetes, and obesity. Patients without OSA had a higher frequency of cerebrovascular disease, peripheral vascular disease, and valvular heart disease. Both groups had a similar prevalence of hypertension and smoking.

### In-hospital mortality and outcomes

The multivariate logistic regression analysis results comparing outcomes in patients with HFpEF with and without OSA are presented in **Table 2**. The results of the primary analysis indicated that patients with OSA had increased mortality (odds ratio [OR], 1.33; 95% confidence interval [CI], 1.28–1.37; *P* < .001), longer length of hospitalization (5.6 vs. 5.3 days; *P* < .001), and increased medical costs of hospitalization ($61,844 vs. $56,182; *P* < .001) compared to patients without OSA. The secondary analysis was a comparison of the prevalence of arrhythmias in both groups. Patients with HFpEF and OSA had a higher prevalence of atrial fibrillation (OR, 1.29; 95% CI, 1.27–1.31; *P* < .001), atrial flutter (OR, 1.13; 95% CI, 1.09–1.17; *P* < .001), premature depolarization (OR, 1.08; 95% CI, 1.01–1.15; *P* < .02), sick sinus syndrome (OR, 1.2; 95% CI, 1.12–1.29; *P* < .001), SVT (OR, 1.07; 95% CI, 1.02–1.13; *P* < .02), ventricular tachycardia (OR, 1.19; 95% CI, 1.13–1.24; *P* < .001), first-degree atrioventricular block (OR, 1.17; 95% CI, 1.09–1.25; *P* < .002), and third-degree atrioventricular block (OR, 1.16; 95% CI, 1.01–1.33; *P* < .03). We also performed a subgroup analysis, comparing the occurrence of arrhythmias with the utilization of inpatient nocturnal CPAP in patients with HFpEF and OSA. The results of this analysis are presented in **Table 3**. Our analysis showed that nocturnal CPAP is not associated with a change in arrhythmia prevalence in the inpatient setting. However, there was a non-significant trend toward a decreased likelihood of cardiac arrest in patients who utilized nocturnal CPAP (0.7% vs. 1.3%; *P* = .06).

## Discussion

This propensity-matched, retrospective study drew data from the 2016–2018 NIS, the largest all-payer inpatient database in the U.S., to compare adverse clinical outcomes in HFpEF patients with and without OSA. The principal findings of this study were as follows: (1) OSA was associated with increased in-hospital mortality, a longer duration of hospitalization, and increased medical costs of hospitalization; (2) OSA was associated with an increased prevalence of atrial fibrillation, atrial flutter, premature depolarization, sick sinus syndrome, SVT, ventricular tachycardia, first-degree atrioventricular block, and third-degree atrioventricular block; and (3) the utilization of inpatient nocturnal CPAP was not associated with a reduced prevalence of arrhythmia.

This study demonstrated that 20% of patients with HFpEF had OSA, consistent with previous reports showing an average prevalence of 23% in men and women in the general population.^3,4^ However, these results were considerably lower than those reported in smaller observational studies, as OSA has been seen in up to 50% of heart failure patients.^22–24^ In agreement with previous reports, OSA was more common in men, those with increased BMI, and those with multiple cardiovascular comorbidities.^22,23,25–27^ Furthermore, previous studies assessing chronic heart failure patients with long-term follow-up have shown OSA to be associated with increased mortality.^28,29^ Moreover, a similar study to the present one utilizing the NIS identified pulmonary hypertension as a strong independent predictor of in-hospital mortality; although OSA was not specifically assessed,^30^ there is considerable overlap in these patient populations.^31,32^

Several pathophysiological mechanisms contribute to the increased mortality and arrhythmogenesis in heart failure patients with OSA. Periods of intermittent hypoxia in OSA result in oxidative stress to cardiac tissues, resulting in impaired energy metabolism and the generation of pro-inflammatory cytokines and reactive oxygen species (ROS).^33^ Furthermore, OSA results in tissue remodeling, inflammation, and autonomic imbalance.^34^ Heart failure in itself is an independent enhancer of ROS production,^35^ tissue remodeling,^36^ inflammation,^37^ and autonomic imbalance,^38^ thereby increasing the propensity for arrhythmogenesis in OSA patients. Autonomic imbalances in OSA are caused by negative thoracic pressure during periods of hypopnea, resulting in alternations in baroreceptor and chemoreceptor reflexes.^39^ These derangements can favor either profound vagal activity, leading to bradyarrhythmia, or sympathetic activation favoring tachyarrhythmias.^40^ This cascade deranges cardiomyocyte ion exchange through action on sodium channels, potassium channels, and gap junction proteins, generating inappropriate action potentials and triggering arrhythmia.^41–43^

Our study found that atrial fibrillation was present in 49% of the patients with HFpEF and OSA, with statistically significant increased odds compared to patients without OSA. While not as common as atrial fibrillation, other tachyarrhythmias, such as atrial flutter, premature depolarization, SVT, and ventricular tachycardia were also more common in patients with OSA. Although not specifically considering HFpEF patients, studies of OSA patients have reported an increased likelihood of tachyarrhythmias.^14,44,45^ Furthermore, OSA is a predictor of the incidence of atrial fibrillation^46^ and its recurrence after cardioversion.^16^ There is a paucity of data regarding atrial flutter. Studies have reported a higher prevalence of atrial flutter in patients with central sleep apnea and Cheyne–Stokes breathing but not so with OSA.^14,47^ Our study also found a higher prevalence of bradyarrhythmias with OSA in HFpEF patients, including sick sinus syndrome, first-degree atrioventricular block, and third-degree atrioventricular block. While not studied in HFpEF patients, studies have commonly documented long pauses with sinus arrest or complete heart block occurring in OSA patients during sleep.^11,13,48^ Interestingly, in 80% of OSA patients with atrioventricular block, sinus node function was normal on electrophysiologic study and reversible with atropine, suggesting normal conduction anatomy in these patients.^49^ Moreover, OSA is 10 times more prevalent in sick sinus syndrome patients compared to the general population.^50^

A subgroup analysis in patients with HFpEF and OSA identified that only 9% of patients had inpatient nocturnal CPAP utilized. We also found that CPAP was not associated with a difference in cardiac conduction disorders; however, there was a non-significant trend toward a decrease in cardiac arrest. Multiple studies have demonstrated the benefits of CPAP in OSA patients in reducing tachyarrhythmias^51,52^ and bradyarrhythmias.^11,48,53^ In addition, CPAP therapy has been shown to improve heart rate variability and baroreflex sensitivity in heart failure patients, thus enhancing vagal modulation and autonomic nervous system regulation.^54,55^ However, there is a paucity of studies assessing the prevalence of arrhythmia in heart failure patients treated with CPAP. One study reported a 59% decrease in the frequency of premature ventricular depolarization.^19^ We hypothesize that no benefit was found with arrhythmia prevalence in our patients because the benefits of CPAP are typically seen with long-term compliance rather than in the short term. However, the trend toward decreased cardiac arrest with CPAP is promising and may signal the potential for short-term benefits of inpatient therapy.

The limitations of this study include the inherent deficiencies of national registry analyses; the NIS is an administrative database susceptible to documentation errors, coding errors, and misdiagnoses. Discharge-level coding relies on individual institutions; therefore, results may not be consistent across various centers. There was likely a degree of misclassification as some patients may have had undiagnosed OSA and were potentially allocated to the incorrect group. In addition, the pathogenesis of bradyarrhythmia with OSA involves vagotonia; therefore, it was anticipated that OSA treatment with CPAP would reduce vagotonia, thus decreasing the arrhythmia risk. However, no difference was detected, indicating a high likelihood for miscategorized OSA status in the NIS database. In addition, our results were likely subject to confounding factors indiscernible by the NIS database, such as New York Heart Association (NYHA) functional classification, grade of diastolic dysfunction, and OSA severity. Specifically, in patients with HFpEF, the diastolic dysfunction grade correlates with increased apnea–hypopnea index, conferring a worse disease burden.^56^ Furthermore, although not assessed in patients with OSA, worsening NYHA functional class has been established as a cause of increased mortality in heart failure patients.^57,58^ Therefore, future prospective studies should categorize patients based on disease severity. Moreover, the NIS cannot capture readmissions; accordingly, there is likely a degree of duplicated data as patients with heart failure have an estimated 30% readmission rate within 90 days.^59^

## Conclusion

The present study demonstrated that OSA in HFpEF patients was associated with increased in-hospital mortality, length of hospitalization, and increased medical costs compared to those without OSA. Furthermore, this analysis showed that OSA in HFpEF patients was associated with an increased likelihood of atrial fibrillation, atrial flutter, premature depolarization, sick sinus syndrome, SVT, ventricular tachycardia, and atrioventricular block. These results were consistent with previous reports in OSA patients without heart failure; however, future studies of heart failure patients with reduced and preserved ejection fraction, respectively, are needed. Furthermore, the use of nocturnal CPAP in these patients was not associated with decreased arrhythmias, although it was only utilized in 9% of patients. However, we found a non-significant trend toward decreased cardiac arrest in patients treated with CPAP, implying a possible benefit of inpatient CPAP.

## Figures and Tables

**Table 1:** Baseline Characteristics for Patients in the Unmatched and Matched Cohorts

	Unmatched Cohort			Matched Cohort		
	HFpEF with OSA	HFpEF Without OSA		HFpEF with OSA	HFpEF Without OSA	
n (unweighted)	25,529	102,244		24,318	24,318	
n (weighted)	127,645	511,220		121,590	121,590	
	**Mean (SD)**	**Mean (SD)**	***P* Value**	**Mean (SD)**	**Mean (SD)**	***P* Value**
Age, years	69.8 (0.1)	75.8 (0.1)	<.001	69.9 (0.1)	69.7 (0.1)	.102
	Weighted n (%)	Weighted n (%)		Weighted n (%)	Weighted n (%)	
Gender						
Male	63,290 (50)	193,145 (38)	<.001	59,435 (49)	58,670 (48)	.17
Female	64,355 (50)	318,075 (62)		62,155 (51)	62,920 (52)	
Race						
Caucasian	91,765 (73)	368,645 (73)	<.001	89,145 (73)	89,650 (73)	.59
African American	21,310 (17)	68,640 (14)		20,710 (17)	20,745 (17)	
Hispanic	7,910 (6)	39,845 (8)		7,750 (6)	7,485 (6)	
Asian	1,425 (1)	11,655 (2)		1,405 (1)	1,225 (1)	
Native American	520 (0.4)	2,260 (0.4)		505 (0.4)	530 (0.4)	
Other	2,100 (2)	11,860 (2)		2,075 (1.7)	1,955 (1.6)	
Hospital teaching status						
Rural	1,225 (10)	55,170 (11)	<.001	11,420 (9)	112 (9)	.17
Urban non-teaching	25,835 (20)	114,915 (23)		24,915 (20)	23,860 (20)	
Urban teaching	89,560 (70)	341,145 (67)		85,255 (70)	86,455 (71)	
Hospital bed size						
Small	25,655 (20)	112,220 (22)	<.001	24,585 (20)	24,085 (20)	.40
Medium	39,365 (31)	160,755 (31)		37,730 (31)	37,215 (31)	
Large	62,630 (49)	238,255 (47)		59,275 (49)	60,290 (50)	
Median income						
$1–$45,999	37,065 (29)	143,395 (29)	.005	36,110 (30)	35,770 (30)	.91
$46,000–$58,999	34,640 (28)	137,170 (27)		33,130 (27)	33,200 (27)	
$59,000–$78,999	30,360 (24)	120,750 (24)		29,185 (24)	29,170 (24)	
$79,000+	23,800 (19)	102,295 (20)		23,165 (19)	23,450 (19)	
Comorbidities (%)						
Cerebrovascular disease	3,015 (2)	15,880 (3)	<.001	2,880 (2)	2,720 (2)	.36
Chronic kidney disease	63,970 (50)	233,240 (46)	<.001	60,890 (50)	60,590 (50)	.59
Chronic obstructive pulmonary disease	56,215 (44)	162,555 (32)	<.001	52,940 (43)	52,300 (43)	.27
Coronary artery disease	59,880 (47)	219,230 (43)	<.001	56,775 (47)	56,545 (47)	.69
Diabetes	76,240 (60)	221,935 (43)	<.001	72,215 (59)	72,445 (60)	.67
Hypertension	79,490 (62)	319,080 (62)	.70	75,735 (62)	76,675 (63)	.09
Obesity	73,895 (58)	11,355 (22)	<.001	69,700 (57)	69,125 (57)	.32
Peripheral vascular disease	12,270 (10)	55,900 (11)	<.001	11,690 (10)	11,240 (9)	.17
Smoking	13,535 (11)	52,775 (10)	.22	13,005 (11)	12,705 (10)	.39
Valvular heart disease	28,050 (22)	135,805 (27)	<.001	26,850 (22)	25,880 (21)	.04

*Abbreviations:* HFpEF, heart failure with preserved ejection fraction; OSA, obstructive sleep apnea; SD, standard deviation.

**Table 2:** In-hospital Outcomes in the Matched and Unmatched Cohorts

	Unmatched Cohort			Matched Cohort		
	HFpEF with OSA	HFpEF Without OSA		HFpEF with OSA	HFpEF Without OSA	
	Mean (SD)	Mean (SD)	*P* Value	Mean (SD)	Mean (SD)	*P* Value
Length of hospitalization, days	5.26 (0.02)	5.31 (.04)	0.002*	5.6 (0.04)	5.3 (0.04)	<0.001*
Medical cost of hospitalization	$56,556 (838)	$56,220 (855)	<0.001*	$61,844 (982)	$56,182 (865)	<0.001*
	**Weighted n (%)**	**Weighted n (%)**	***P* Value**	**Weighted n (%)**	**Weighted n (%)**	**Odds Ratio (95% CI); *P* Value)**
In-hospital mortality	10,775 (2.1)	1,510 (1.2)	<0.001*	2,095 (1.7)	1,405 (1.2)	1.33 (1.28–1.37); <0.001*
Atrial fibrillation	62,760 (49.2)	248,625 (48.6)	0.161	60,120 (49.4)	52,485 (43.2)	1.29 (1.27–1.31); <0.001*
Atrial flutter	6,715 (5.3)	22,215 (4.3)	<0.001*	6,425 (5.3)	5,735 (4.7)	1.13 (1.09–1.17); <0.001*
Cardiac arrest	1,060 (0.8)	3,980 (.8)	0.423	990 (0.8)	1,020 (0.8)	0.97 (0.89–1.06); 0.502
Premature depolarization	2,000 (1.6)	7,390 (1.4)	0.152	1,910 (1.6)	1,770 (1.5)	1.08 (1.01–1.15); 0.02*
Sick sinus syndrome	1,770 (1.4)	7,810 (1.5)	0.088	1,715 (1.4)	1,435 (1.2)	1.2 (1.12–1.29); <0.001*
Supraventricular tachycardia	10,070 (2.0)	2,340 (1.8)	0.135	2,420 (2.0)	2,265 (1.9)	1.07 (1.02–1.13); 0.022*
Ventricular tachycardia	1,650 (1.3)	7,610 (1.5)	0.016*	1,910 (1.6)	1,545 (1.3)	1.19 (1.13–1.24); <0.001*
Ventricular fibrillation	180 (0.1)	710 (.1)	0.935	205 (0.2)	170 (0.1)	1.17 (1.02–1.36); 0.071
First-degree AV block	1,795 (1.4)	6,405 (1.3)	0.056	1,690 (1.4)	1,580 (1.3)	1.17 (1.09–1.25); 0.002*
Second-degree AV block	655 (0.5)	2,250 (.4)	0.136	600 (0.5)	590 (0.5)	1.02 (0.91–1.14); 0.771
Third-degree AV block	460 (0.4)	2,135 (.4)	0.170	435 (0.4)	375 (0.3)	1.16 (1.01–1.33); 0.03*

*Abbreviations:* AV, atrioventricular; CI, confidence interval; HFpEF, heart failure with preserved ejection fraction; OSA, obstructive sleep apnea; SD, standard deviation. *Statistically significant.

**Table 3:** In-hospital Outcomes in Patients with and Without Nocturnal Continuous Positive Airway Pressure

	Unmatched Cohort			Matched Cohort		
	HFpEF with OSA on Nocturnal CPAP	HFpEF with OSA Without Nocturnal CPAP		HFpEF with OSA on Nocturnal CPAP	HFpEF with OSA Without Nocturnal CPAP	
n (unweighted)	2,250	22,068		2,250	2,250	
n (weighted)	11,250	110,340		11,250	11,250	
	**Weighted n (%)**	**Weighted n (%)**	***P* Value**	**Weighted n (%)**	**Weighted n (%)**	***P* Value**
Atrial fibrillation	2,155 (19.2)	22,440 (20.3)	.185	2,155 (19.2)	2,135 (19)	.879
Atrial flutter	670 (6)	5,755 (5.2)	.204	670 (6)	570 (5.1)	.236
Cardiac arrest	150 (1.3)	840 (0.8)	.024	79 (0.7)	150 (1.3)	.063
Premature depolarization	225 (2)	1,685 (1.5)	.136	225 (2)	150 (1.3)	.090
Supraventricular tachycardia	245 (2.2)	2,020 (1.8)	.274	245 (2.2)	230 (2)	.751
Ventricular tachycardia	160 (1.4)	1,385 (1.3)	.514	160 (1.4)	135 (1.2)	.412
Ventricular fibrillation	25 (0.2)	145 (0.1)	.371	25 (0.2)	0 (0)	NA
First-degree AV block	195 (1.7)	1,495 (1.4)	.224	195 (1.7)	185 (1.6)	.824
Second-degree AV block	60 (0.5)	540 (0.5)	.799	60 (0.5)	35 (0.3)	.273
Third-degree AV block	30 (0.3)	405 (0.4)	.379	30 (0.3)	60 (0.5)	.176

*Abbreviations:* AV, atrioventricular; CI, confidence interval; HFpEF, heart failure with preserved ejection fraction; NA, not available; OSA, obstructive sleep apnea; SD, standard deviation.

## References

[1] Gladden JD, Linke WA, Redfield MM (2014). Heart failure with preserved ejection fraction. Pflugers Arch.

[2] Borlaug BA, Redfield MM (2011). Diastolic and systolic heart failure are distinct phenotypes within the heart failure spectrum. Circulation.

[3] Young T, Palta M, Dempsey J, Peppard PE, Nieto FJ, Hla KM (2009). Burden of sleep apnea: rationale, design, and major findings of the Wisconsin Sleep Cohort Study. WMJ.

[4] Peppard PE, Young T, Barnet JH, Palta M, Hagen EW, Hla KM (2013). Increased prevalence of sleep-disordered breathing in adults. Am J Epidemiol.

[5] Herrscher TE, Akre H, Øverland B, Sandvik L, Westheim AS (2011). High prevalence of sleep apnea in heart failure outpatients: even in patients with preserved systolic function. J Card Fail.

[6] Yeghiazarians Y, Jneid H, Tietjens JR (2021). Obstructive sleep apnea and cardiovascular disease a scientific statement from the American Heart Association. Circulation.

[7] Gami AS, Olson EJ, Shen WK (2013). Obstructive sleep apnea and the risk of sudden cardiac death: a longitudinal study of 10,701 adults. J Am Coll Cardiol.

[8] Gami AS, Howard DE, Olson EJ, Somers VK (2005). Day-night pattern of sudden death in obstructive sleep apnea. N Engl J Med.

[9] Tavares L, Lador A, Valderrábano M (2021). Sleep apnea and atrial fibrillation: role of the cardiac autonomic nervous system. Methodist Debakey Cardiovasc J.

[10] Patel N, Donahue C, Shenoy A, Patel A, El-Sherif N (2017). Obstructive sleep apnea and arrhythmia: a systemic review. Int J Cardiol.

[11] Simantirakis EN, Schiza SI, Marketou ME (2004). Severe bradyarrhythmias in patients with sleep apnoea: the effect of continuous positive airway pressure treatment: a long-term evaluation using an insertable loop recorder. Eur Heart J.

[12] Flemons WW, Remmers JE, Gillis AM (1993). Sleep apnea and cardiac arrhythmias. Is there a relationship?. Am Rev Respir Dis.

[13] Becker HF, Koehler U, Stammnitz A, Peter JH (1998). Heart block in patients with sleep apnoea. Thorax.

[14] Mehra R, Stone KL, Varosy PD (2009). Nocturnal arrhythmias across a spectrum of obstructive and central sleep-disordered breathing in older men: outcomes of sleep disorders in older men (MrOS sleep) study. Arch Intern Med.

[15] Raghuram A, Clay R, Kumbam A, Tereshchenko LG, Khan A (2014). A systematic review of the association between obstructive sleep apnea and ventricular arrhythmias. J Clin Sleep Med.

[16] Kanagala R, Murali NS, Friedman PA (2003). Obstructive sleep apnea and the recurrence of atrial fibrillation. Circulation.

[17] Naruse Y, Tada H, Satoh M (2013). Concomitant obstructive sleep apnea increases the recurrence of atrial fibrillation following radiofrequency catheter ablation of atrial fibrillation: clinical impact of continuous positive airway pressure therapy. Heart Rhythm.

[18] Holmqvist F, Guan N, Zhu Z (2015). Impact of obstructive sleep apnea and continuous positive airway pressure therapy on outcomes in patients with atrial fibrillation—results from the Outcomes Registry for Better Informed Treatment of Atrial Fibrillation (ORBIT-AF). Am Heart J.

[19] Ryan CM, Usui K, Floras JS, Bradley TD (2005). Effect of continuous positive airway pressure on ventricular ectopy in heart failure patients with obstructive sleep apnoea. Thorax.

[20] Doherty LS, Kiely JL, Swan V, McNicholas WT (2005). Long-term effects of nasal continuous positive airway pressure therapy on cardiovascular outcomes in sleep apnea syndrome. Chest.

[21] Houchens RL, Ross D EA HCUP Methods Series Using the HCUP National Inpatient Sample to Estimate Trends (Revised 12/15/15) Report # 2006-05.

[22] Bitter T, Faber L, Hering D, Langer C, Horstkotte D, Oldenburg O (2009). Sleep-disordered breathing in heart failure with normal left ventricular ejection fraction. Eur J Heart Fail.

[23] Chan J, Sanderson J, Chan W (1997). Prevalence of sleep-disordered breathing in diastolic heart failure. Chest.

[24] Kasai T, Bradley TD (2011). Obstructive sleep apnea and heart failure: pathophysiologic and therapeutic implications. J Am Coll Cardiol.

[25] Abdullah A, Eigbire G, Salama A, Wahab A, Nadkarni N, Alweis R (2018). Relation of obstructive sleep apnea to risk of hospitalization in patients with heart failure and preserved ejection fraction from the national inpatient sample. Am J Cardiol.

[26] Mentz RJ, Kelly JP, von Lueder TG (2014). Noncardiac comorbidities in heart failure with reduced versus preserved ejection fraction. J Am Coll Cardiol.

[27] Sin DD, Fitzgerald F, Parker JD, Newton G, Floras JS, Bradley TD (1999). Risk factors for central and obstructive sleep apnea in 450 men and women with congestive heart failure. Am J Respir Crit Care Med.

[28] Khayat R, Jarjoura D, Porter K (2015). Sleep disordered breathing and post-discharge mortality in patients with acute heart failure. Eur Heart J.

[29] Damy T, Margarit L, Noroc A (2012). Prognostic impact of sleep-disordered breathing and its treatment with nocturnal ventilation for chronic heart failure. Eur J Heart Fail.

[30] Goyal P, Almarzooq ZI, Horn EM (2016). Characteristics of hospitalizations for heart failure with preserved ejection fraction. Am J Med.

[31] Ismail K, Roberts K, Manning P, Manley C, Hill NS (2015). OSA and pulmonary hypertension: time for a new look. Chest.

[32] Minai OA, Ricaurte B, Kaw R (2009). Frequency and impact of pulmonary hypertension in patients with obstructive sleep apnea syndrome. Am J Cardiol.

[33] May AM, Mehra R (2014). Obstructive sleep apnea: role of intermittent hypoxia and inflammation. Semin Respir Crit Care Med.

[34] Chadda KR, Fazmin IT, Ahmad S (2018). Arrhythmogenic mechanisms of obstructive sleep apnea in heart failure patients. Sleep.

[35] Tsutsui H, Kinugawa S, Matsushima S (2011). Oxidative stress and heart failure. Am J Physiol Heart Circ Physiol.

[36] Nikolaidou T, Cai XJ, Stephenson RS (2015). Congestive heart failure leads to prolongation of the pr interval and atrioventricular junction enlargement and ion channel remodelling in the rabbit. PLoS One.

[37] Askevold ET, Gullestad L, Dahl CP, Yndestad A, Ueland T, Aukrust P (2014). Interleukin-6 signaling, soluble glycoprotein 130, and inflammation in heart failure. Curr Heart Fail Rep.

[38] Florea VG, Cohn JN (2014). The autonomic nervous system and heart failure. Circ Res.

[39] Smith RP, Veale D, Pépin JL, Lévy PA (1998). Obstructive sleep apnoea and the autonomic nervous system. Sleep Med Rev.

[40] Leung RST (2009). Sleep-disordered breathing: autonomic mechanisms and arrhythmias. Prog Cardiovasc Dis.

[41] Jeong EM, Liu M, Sturdy M (2012). Metabolic stress, reactive oxygen species, and arrhythmia. J Mol Cell Cardiol.

[42] Köhler AC, Sag CM, Maier LS (2014). Reactive oxygen species and excitation-contraction coupling in the context of cardiac pathology. J Mol Cell Cardiol.

[43] Kozhevnikov DO, Yamamoto K, Robotis D, Restivo M, El-Sherif N (2002). Electrophysiological mechanism of enhanced susceptibility of hypertrophied heart to acquired torsade de pointes arrhythmias: tridimensional mapping of activation and recovery patterns. Circulation.

[44] Mehra R, Benjamin EJ, Shahar E (2006). Association of nocturnal arrhythmias with sleep-disordered breathing: the Sleep Heart Health Study. Am J Respir Crit Care Med.

[45] Gami AS, Pressman G, Caples SM (2004). Association of atrial fibrillation and obstructive sleep apnea. Circulation.

[46] Gami AS, Hodge DO, Herges RM (2007). Obstructive sleep apnea, obesity, and the risk of incident atrial fibrillation. J Am Coll Cardiol.

[47] Anzai T, Grandinetti A, Katz AR, Hurwitz EL, Wu YY, Masaki K (2020). Association between central sleep apnea and atrial fibrillation/flutter in Japanese-American men: The Kuakini Honolulu Heart Program (HHP) and Honolulu-Asia Aging Study (HAAS). J Electrocardiol.

[48] Becker H, Brandenburg U, Peter JH, Von Wichert P (1995). Reversal of sinus arrest and atrioventricular conduction block in patients with sleep apnea during nasal continuous positive airway pressure. Am J Respir Crit Care Med.

[49] Grimm W, Hoffmann J, Menz V (1996). Electrophysiologic evaluation of sinus node function and atrioventricular conduction in patients with prolonged ventricular asystole during obstructive sleep apnea. Am J Cardiol.

[50] Martí Almor J, Félez Flor M, Balcells E, Cladellas M, Broquetas J, Bruguera J (2006). Prevalence of obstructive sleep apnea syndrome in patients with sick sinus syndrome. Rev Esp Cardiol (English Ed).

[51] Abe H, Takahashi M, Yaegashi H (2010). Efficacy of continuous positive airway pressure on arrhythmias in obstructive sleep apnea patients. Heart Vessels.

[52] Varga PC, Rosianu HS, Vesa ŞC, Hancu BGD, Beyer R, Pop CM (2020). The impact of continuous positive airway pressure on cardiac arrhythmias in patients with sleep apnea. J Res Med Sci.

[53] Grimm W, Koehler U, Fus E (2000). Outcome of patients with sleep apnea-associated severe bradyarrhythmias after continuous positive airway pressure therapy. Am J Cardiol.

[54] Gilman MP, Floras JS, Usui K, Kaneko Y, Leung RST, Bradley TD (2008). Continuous positive airway pressure increases heart rate variability in heart failure patients with obstructive sleep apnoea. Clin Sci (Lond).

[55] Ruttanaumpawan P, Gilman MP, Usui K, Floras JS, Bradley TD (2008). Sustained effect of continuous positive airway pressure on baroreflex sensitivity in congestive heart failure patients with obstructive sleep apnea. J Hypertens.

[56] Gupta N, Agrawal S, Goel AD, Ish P, Chakrabarti S, Suri JC (2020). Profile of sleep disordered breathing in heart failure with preserved ejection fraction. Monaldi Arch Chest Dis.

[57] Chioncel O, Lainscak M, Seferovic PM (2017). Epidemiology and one-year outcomes in patients with chronic heart failure and preserved, mid-range and reduced ejection fraction: an analysis of the ESC Heart Failure Long-Term Registry. Eur J Heart Fail.

[58] Pocock SJ, Wang D, Pfeffer MA (2006). Predictors of mortality and morbidity in patients with chronic heart failure. Eur Heart J.

[59] Khan MS, Sreenivasan J, Lateef N (2021). Trends in 30- and 90-day readmission rates for heart failure. Circ Hear Fail.

